# Tactile Perception of Roughness and Hardness to Discriminate Materials by Friction-Induced Vibration

**DOI:** 10.3390/s17122748

**Published:** 2017-11-28

**Authors:** Shuyang Ding, Yunlu Pan, Mingsi Tong, Xuezeng Zhao

**Affiliations:** Key Laboratory of Micro-Systems and Micro-Structures Manufacturing, Ministry of Education and School of Mechatronics Engineering, Harbin Institute of Technology, Harbin 150001, China; ledaodsy@126.com (S.D.); tongms@hit.edu.cn (M.T.)

**Keywords:** tactile perception, materials discrimination, tactile sensor, friction induced vibration, surface roughness, hardness

## Abstract

The human fingertip is an exquisitely powerful bio-tactile sensor in perceiving different materials based on various highly-sensitive mechanoreceptors distributed all over the skin. The tactile perception of surface roughness and material hardness can be estimated by skin vibrations generated during a fingertip stroking of a surface instead of being maintained in a static position. Moreover, reciprocating sliding with increasing velocities and pressures are two common behaviors in humans to discriminate different materials, but the question remains as to what the correlation of the sliding velocity and normal load on the tactile perceptions of surface roughness and hardness is for material discrimination. In order to investigate this correlation, a finger-inspired crossed-I beam structure tactile tester has been designed to mimic the anthropic tactile discrimination behaviors. A novel method of characterizing the fast Fourier transform integral (FFT) slope of the vibration acceleration signal generated from fingertip rubbing on surfaces at increasing sliding velocity and normal load, respectively, are defined as *k_v_* and *k_w_*, and is proposed to discriminate the surface roughness and hardness of different materials. Over eight types of materials were tested, and they proved the capability and advantages of this high tactile-discriminating method. Our study may find applications in investigating humanoid robot perceptual abilities.

## 1. Introduction

Tactile perception through finger touch plays an essential role in our interaction with the external environment. A dynamic process involving movement, such as repetitively stroking a surface to assess its texture or roughness, pressing throughout a material to explore its hardness, is highly needed to access the nature of materials we are in contact with throughout our daily lives [1]. In fact, it became possible to discriminate similar textures by fingertip sliding on textured surfaces [2,3,4]. Fingerprint patterns enable the perception of a surface’s fine texture by amplifying the vibration signals during a fingertip scanning over the surface [5,6,7,8]. The interlocked microstructures between the epidermal and dermal layers of the skin are supposed to be functional amplifiers and efficiently transfer the tactile stimulation to various cutaneous mechanoreceptors [9,10]. Within human skin, slowly-adapting (SA) receptors, such as Merkel and Ruffini corpuscles, respond to sustained touch and pressure on the skin, and rapid-adapting (RA) receptors, for example, Meissner and Pacinian corpuscles, respond to dynamic touch and vibration stimuli [11,12,13,14]. All of these mechanoreceptors enable humans to precisely perceive and discriminate mechanical and thermal stimuli. These mechanics of tactile sensing provide cues about the physical properties of touched objects, such as shape, softness, frictional properties, and surface topography, as the sense of touch [15,16,17,18,19]. Until recently, studies in the field of tactile perception and material discrimination have generally focused on one or two materials assumed to be discriminated on the basis of their physical properties, such as frictional characteristics [20,21], surface textures [22,23,24], material hardness [25], or thermal properties [26,27]. However, it has never been suggested that different materials we touched could be discriminated by tactile perception of surface roughness and material hardness, which was evaluated by friction-induced vibrations generated during a fingertip interacting with the material surfaces. This material discrimination study is a further exploration of a simple, non-intrusive, and high tactile-discriminating approach to reveal the secrets of human tactile sensing in material discrimination.

### 1.1. Material Tactile Discrimination

Surface texture and material hardness are two essential properties of a material and commonly recognized to conduct a material identification. Most previously studied surface-related tactile perceptual tasks rely on fingertip sliding on surfaces to discriminate materials with frictional cues [28]. Textured surfaces with wrinkle wavelengths ranging from 300 nm to 90 µm were correlated with the finger friction coefficient, and the frictional cues illustrated that human tactile discrimination extends to the nanoscale with the lowest amplitude of the ridges being approximately 10 nm [29]. The primary reason for these discrimination performances is that surface mechanical fluctuations induced by fingertip sliding not only reveal the topography information of a surface, but also present interfacial deformations induced by static/dynamic contact [6]. A study of the tactile perception of bathroom tissues by a tribo-acoustic artificial finger sensor, measuring friction and vibrations during the sliding of an artificial finger on the surface of the tissues, proved that both the surface texture and material softness have good correlations with friction-induced vibrations, and a principal component analysis classified the bathroom tissues into four different groups [28]. In recent years, surface roughness, as one of the most important physical characteristics in surface texture, has been widely investigated by frictional properties and vibration frequency spectrum analysis [30,31,32,33,34,35,36,37,38,39]. However, it has never been suggested that the differences between the materials we touch could be discriminated by tactile perception of surface roughness and material hardness as a function of sliding velocity and pressing normal load, which is inspired from the nature of fingertip identification of the materials.

**Figure 1:**
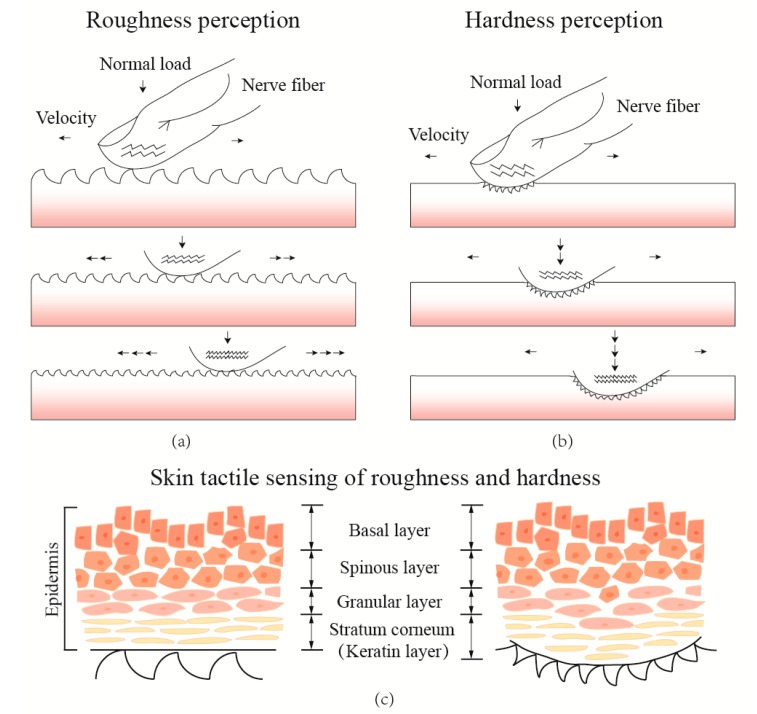
(**a**) Schematic illustrating roughness tactile perception based on the skin vibration as a function of sliding velocity during a fingertip rubbing on different textured surfaces; (**b**) hardness tactile perception as a function of normal load on various hardness materials; and (**c**) the structure of skin epidermis and its deformation in both two tactile perceptions.

Complex mechanical fluctuations take place, as well as the transitory phases between sticking and slipping, during the fingertip scanning of a material’s surfaces for a range of rubbing velocities and loads. As shown in Figure 1, when the fingertip reciprocatingly rubs the material’s surface with increasing rubbing velocity and pressing normal loads, various vibration signals are detected by the mechanoreceptors distributed in the skin dermis of the fingertip [14] and are conducted to the nerve fibers, then finally recognized by the human brain [40]. Figure 1a,b presents a simplified schematic of human roughness and hardness tactile perceptions, which illustrate the correlation of surface textures and interfacial deformations as a function of sliding velocity and normal load at the interface between the fingertip and the material surface. The skin structure of the epidermis layer [41], as shown in Figure 1c, presents a schematic of the physical relationship between the fingertip and the material during interaction. The four layers of skin epidermis interact with the surface texture, and vibrations and deformations are generated with complex mechanical fluctuations for human tactile sensing [30,42]. Therefore, in material tactile sensing, the vibration stimuli from the fingertip–surface interaction is highly complex due to the deformation of the finger and the substrate surface, and simultaneously affected by the scanning velocity and pressure [43]. Thus, an investigation into the relationship between material tactile discrimination and the material physical properties is highly desirable to further illuminate the mechanical fluctuations for material tactile sensing of the human fingertip.

### 1.2. Objective of the Paper

In this paper, we study the tactile perception characteristics of vibration signals during an artificial fingertip rubbing on eight kinds of materials with different hardness and manufactured with various roughness. In various rubbing velocity and normal load conditions, the correlation between the frequency spectra integral of the material’s tactile vibration signals and the material’s physical properties are investigated in order to understand how the sliding velocity and normal load characterize the tactile perception of the surface roughness and hardness in human fingertip tactile sensing. We also propose a new material discrimination approach. Here, rubbing is conducted using a fingerprint-patterned artificial fingertip and a low-frequency acceleration sensor relevant for material sliding to collect vibration characteristics.

## 2. Experimental

### 2.1. Material Preparation and Method

For characterizing the tactile discrimination of materials, glass, stainless, aluminum, polymethyl methacrylate (PMMA), epikote (EP), wood, silicone, and polydimethylsiloxane (PDMS) were chosen according to their hardness and manufactured as substrates in the same dimensions (20 mm × 100 mm × 10 mm). There were two categories of these materials used in this tactile discrimination study: surface roughness and material hardness. All of these samples were prepared under the same laboratory conditions (25 °C and relative humidity (RH) 55%) and appropriately cleaned by relevant approaches with ethanol or acetone solutions, as mentioned in a previous study [14].

In the roughness tactile discrimination test, samples with different surface roughness were produced by a commercial sander (FS-100C, Nitto Kohki, Tochigi, Japan). Three glass substrates were manufactured by 200, 600, and 1000 mesh sandpapers (991A, Matador Starcke, Germany), and five aluminum substrates were ground by 60, 120, 220, 400, 800, and 1000 mesh sandpapers. In contrast with these two materials, silicone (Zhermack HD+ A, Badia Polesine, Italy) was compressed on the glass substrates for creating three comparable softer samples with similar surface roughness. Moreover, a virgin pig skin, which was studied in our previous research [14], was prepared and a cooperation test in this section was conducted as well. All surface roughnesses of these samples are shown in the following figures and Table 1 and Table 2.

For the hardness tactile discrimination test, five different mass ratios of PDMS and curing agent (Sylgard 184 silicone elastomer kit, Auburn, AL, USA) mixtures were prepared, respectively, as different mass ratios lead to different hardness [44]. The mixtures were vacuumed for 20 min in a vacuum drier at 25 °C and then heated at 80 °C for 40 min in a lab oven. Finally, five PDMS substrates with five different hardnesses, ranging from 15 HS to 54 HS (in Shore-A), were produced as required.

Furthermore, the eight common materials mentioned above were particularly prepared and tested in a common material tactile discrimination test based on a surface roughness and hardness tactile perception analysis. An initial glass, a commercial aluminum (6061), a PDMS (10:1 mass ratio) substrate, previously manufactured, a silicone sample prepared by being compressed on an initial glass substrate, commercial PMMA (Shengjili Acrylic, Shenzhen, China), EP, 304 stainless steel, and an initial wooden board (pine) were chosen and manufactured in the same dimensions. All eight common materials’ physical properties are presented in Table 2.

In this paper, friction-induced vibration signals were collected by an acceleration sensor, and a novel method of material tactile discrimination was proposed based on a frequency spectrum analysis of the vibration signals generated during an artificial fingertip sliding on the interface of the fingertip and the surfaces with various rubbing velocity and normal load. The fast Fourier transform (FFT) of the friction force and acceleration signals was used in the spectral analysis [45,46]. Based on a large number of experiments’ data frequency analyses, we proposed a new method to distinguish the surface roughness and hardness properties of different materials by the frequency spectral integral of the vibration acceleration signal generated from the interface between the fingertip and the surface, which was defined as *S*(*FFT*) by the following relationship:

(1) $$S\left(FFT\right)=\;\frac{b-a}{2N}\overset{N}{\underset{n=1}{\sum }}\left[FFT\left(f_{n}\right)+FFT\left(f_{n+1}\right)\right]$$

where *S*(*FFT*) is the integral of *FFT*(*f*), *FFT*(*fn*) is the fast Fourier transform of the variable *x*(*t*), *N* is the number of sampling points, and the spacing between each point is equal to the scalar value $\frac{b-a}{N}$.

Then, characterizations of *S*(*FFT*) as a function of the rubbing velocity and normal load, respectively, were conducted in the following surface roughness and hardness tactile discrimination tests in order to determine a simple and reliable method to distinguish materials by tactile perception of roughness and hardness.

### 2.2. Experimental Apparatus and Procedure

**Figure 2:**
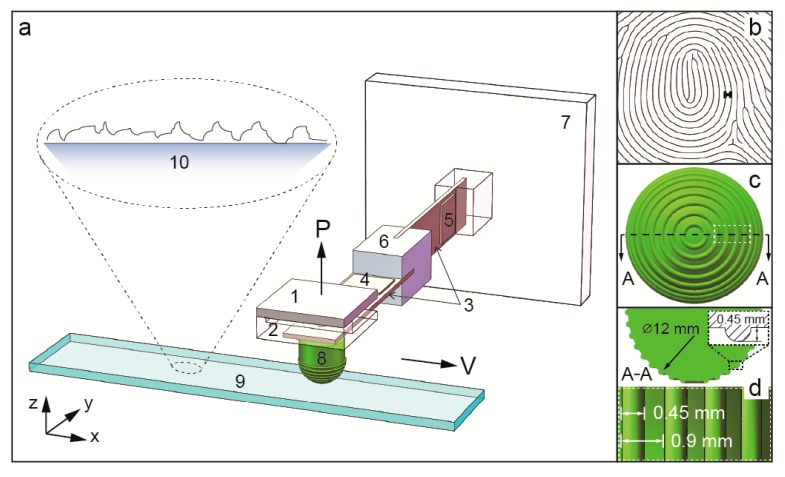
(**a**) Sketch of the experimental setup. A three-axis acceleration sensor (1) is mounted on an aluminum holder (2) based on a pair of crossed-I beams (3) upon which were installed two strain gauge force sensors: the normal load sensor (4) and the friction sensor (5) were connected by two aluminum holders (6) and (7), an artificial fingertip (made of silicone with a height of 15 mm, a diameter of 12 mm, a hardness of 55 Shore-A, and a surface roughness (Ra) of 0.24 µm) whose surface is patterned with parallel ridges (an artificial cyclic fingerprint with semicircle ridges at a height of 0.45 mm and width of 0.45 mm) to mimic a human fingerprint as shown in (**b**–**d**). The fingertip probe slides on a 200 mesh ground glass substrate (9) and the surface presented a non-Gaussian random rough surface (10).

In this study, a crossed I-beam structure tactile tribometer was designed with a fingerprint-patterned artificial fingertip mounted at the end of a flexible cantilever beam, which was inspired by the bio-structures of the human finger conducting touch-sensing as shown in Figure 2.

The system’s friction-induced vibration was measured by a small, low profile (4 mm × 4 mm × 1.45 mm, mass 1.27 g), three-dimensional (3D) capacitive-type acceleration sensor (Analog Devices ADXL335, Norwood, NJ, USA), which was screwed on a sensor holder at the edge of the cantilever beam as shown in Figure 2a. The sensor uses three sets of cantilever beams inside the housing to provide measurements along three axes. The accelerometer has a full-scale range of ±3 g, and the bandwidth has a range of 0.5 Hz–1.6 kHz in the x and y directions, 0.5–550 Hz in the z direction, and a sensitivity at x, y, and z of 300 ± 0.001 mV/g. The natural frequency of the beams was 5 kHz above the measured frequency ranging from 0 to 500 Hz. Normal load and friction force were measured with strain gauge sensors mounted on the edge of the beams with a gauge factor of 115 used. The artificial fingertip, as shown in Figure 2a, was loaded against a reciprocating substrate that was mounted on a commercial three-axis adjustable stage. The beam structure was mounted on a two-axis linear stage (Newport 462 series YZ axis, Irvine, CA, USA). The normal load was applied by a micrometer, which was installed on the top of the two-axis linear stage. A laboratory jack was used to lift the entire stage in the vertical direction. A reciprocating platform (THK1505) was used to provide reciprocating motion. A Newport XYZ axis stage was installed on the reciprocating stage, and a three-axis triangle adjustable sample stage (SQ240-4) was installed on the top to provide the samples with a horizontal position. The reciprocating sliding device we built can provide a linear motion with an adjustable stroke length of up to 200 mm and variable frequency of up to 5 Hz (the velocity is 100 mm/s and the stroke is 10 mm).

Normal load, friction force, and accelerometer data were sampled at 4 kHz, considering that a frequency measurement range of 0–1.6 kHz is sufficient to measure all the dominant frequencies of vibration under the present experimental conditions. These parameters were digitized with a resolution of 12 bits in the range of 3.5 V through Labview 2016 software (National Instrument TM, Austin, TX, USA) and onboard electronics. Then, the spectrum analysis was obtained by taking the FFT and frequency spectral integral of the acceleration data through a program in the MATLAB software package.

For the material tactile discrimination test, all samples were prepared and cleaned by ethanol or acetone, respectively. The artificial finger interacted with a reciprocating sample with a normal load, which was applied to the surfaces through the micrometer actuator from 100 to 400 mN, and the reciprocating sliding velocity ranged from 10 to 50 mm/s, with a constant scanning distance of 50 mm. The roughness (R_a_) of rough materials, such as wood, stainless steel, and aluminum, was measured by a portable surface roughness tester (TR200, TIME, Beijing, China), and smooth materials, such as glass, PMMA, EP, silicone, and PDMS, were measured by an atomic force microscope (Bruker Innova AFM, Camarillo, CA, USA) to provide a higher-precision measurement of roughness with little damage to the surfaces. The hardness of these hard materials, such as glass, PMMA, EP, stainless steel, and aluminum, was measured by an HVS-100 hardness tester, and the soft materials of PDMS, silicone, and wood were measured by Shore Durometer (Handpi LXA, Wenzhou, China), which is applicable to colloid and fabric materials. The average roughness and hardness were obtained by calculating the mean of five measurements for each material. All of these physical properties are listed in Table 1 and Table 2.

## 3. Result and Discussion

### 3.1. Tactile Perception of Glass, Aluminum, Silicone, and Pig Skin

**Figure 3:**
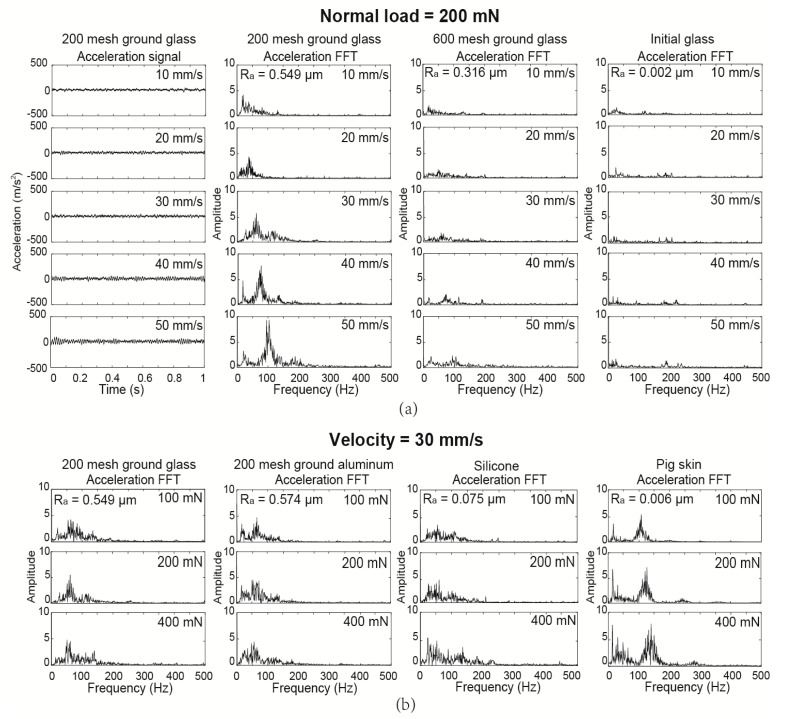
Glass, aluminum, silicone, and pig skin tactile perception (**a**) acceleration signal and frequency analysis (fast Fourier transform (FFT)) of three different roughness glass samples as a function of velocity ranging from 10 to 50 mm/s and a normal load equal to 200 mN; and (**b**) the frequency analysis (FFT) of four different hardness materials as normal load increases from 100 to 400 mN and with a sliding velocity equal to 30 mm/s.

In this section, one 200 mesh sandpaper ground glass sample, one 600 mesh sandpaper ground glass sample, and an initial glass sample were used to conduct the roughness-velocity test with a sliding velocity range of 10–50 mm/s and a normal load of 200 mN, as shown in Figure 3a. The acceleration signals and FFT spectral analysis of the 200 mesh ground glass sample are presented as a sample in the first two columns. The data shows that the amplitude of the acceleration FFT peaks increased and the whole frequency spectrum curves covered a larger area as the sliding velocity increased. A similar trend was obtained on the 600 mesh ground and initial glass samples with increasing sliding velocity. Comparing the FFT frequency spectrum of 200 and 600 mesh ground glass and the initial glass at the same velocity, it shows that the frequency spectrum area decreases as a function of surface roughness. Then, compared with the two smoother samples, the 200 mesh ground glass shows the largest increasing rate of the frequency spectrum area with the sliding velocity increasing from 10 to 50 mm/s.

In comparing with the velocity test above, there are four columns of FFT data shown in Figure 3b, which demonstrate a normal load test on four different materials: a 200 mesh ground glass, a 200 mesh ground aluminum, a 200 mesh glass surface-molded silicone, and a virgin pig skin sample, respectively. All of these samples were tested with a normal load increasing from 100 to 400 mN and a constant velocity of 30 mm/s. The four columns of the FFT analysis for the glass, aluminum, silicone, and pig skin samples show that the frequency spectrum area increases as the material hardness decreases, while with an increasing normal load, soft samples, such as pig skin and silicone, increased in frequency amplitude and spectrum area. The FFT data of the pig skin, which is the softest surface, presents the largest frequency area among all of the materials. From a synthetic analysis of the data, which is shown in Figure 3, it can be inferred that the sliding velocity changing the tactile perception test can determine the surface roughness of the same, or various materials, and the quantified normal load verifying the tactile perception test can distinguish between soft and hard materials. In order to further investigate this proposed theory, a roughness tactile discrimination test and a hardness tactile discrimination test were carried out.

### 3.2. Tactile Discrimination of Aluminum with Different Roughness

**Figure 4:**
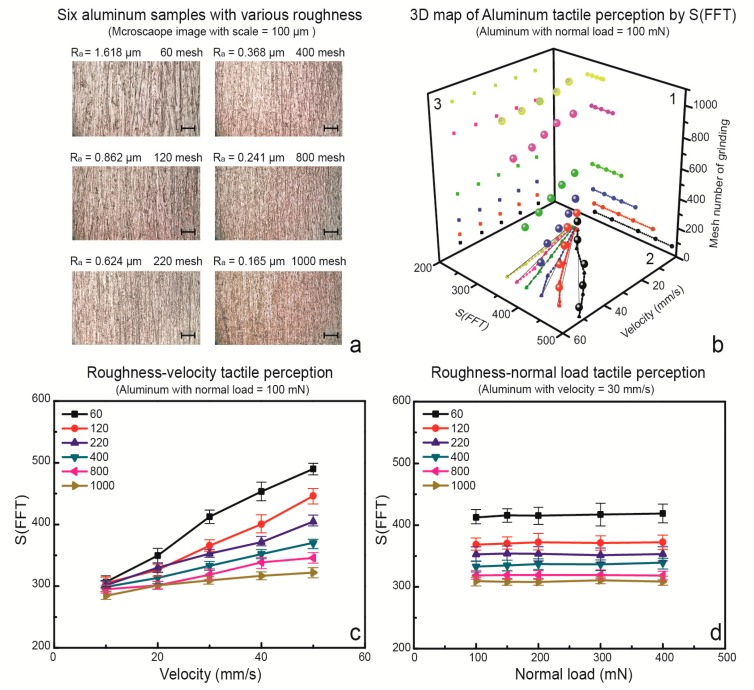
Aluminum roughness tactile discrimination as a function of velocity and normal load. (**a**) Microscope images of six aluminum surface topographies with various roughness; (**b**) three-dimensional (3D) map of aluminum tactile perception by *S*(*FFT*) with a normal load equal to 100 mN; (**c**) Roughness-velocity tactile perception as a function of velocity; (**d**) Roughness-normal load tactile perception as a function of normal load.

Six aluminum samples were ground by sandpapers of 60 to 1000 mesh with a commercial sander, respectively, as shown in Figure 4a. The surface roughness of these three samples were measured, as shown in Table 1. We specifically tested the possibility of surface roughness tactile discrimination by vibration frequency spectra generated by a fingerprint-patterned artificial fingertip reciprocatingly sliding with increasing velocities. A normal load of 100 mN was applied to each roughness tactile test with an increasing velocity from 10 to 50 mm/s. *S*(*FFT*) is the integral of the FFT frequency spectra from 0 to 500 Hz. Then, a 3D map of the aluminum roughness tactile perception, including the *S*(*FFT*), velocity, and mesh number of grinding as x, y, and z axes respectively, is shown in Figure 4b, which presents a fall map of spiral distribution dots. This fall map may have great potential for tactile perception studies on materials with different hardnesses as well. This figure shows the possibility of roughness tactile discrimination based on the *S*(*FFT*) with increasing velocities, as the rougher surface induced a wider range of *S*(*FFT*) values, such as the 60 mesh ground aluminum sample showing a *S*(*FFT*) value range from 286 to 481 when the sliding velocity increases. However, smooth surfaces, such as the 1000 mesh ground aluminum sample, produced a smaller range of *S*(*FFT*), from 267 to 321, as shown in Figure 4b, Section 1. Then, the *S*(*FFT*) of each sample with increasing velocity is shown in Figure 4c. It is obvious that the slopes of each curve for each sample are different, as a larger slope can be expected for rougher surfaces.

The slope values of these curves were defined as *k_v_*, based on the least-squares method, and are listed in Table 1. There is a clear monotonic relationship between *k_v_* and the roughness. Therefore, increasing the scanning velocity of a fingertip sliding on a solid surface could be a way to distinguish a rough surface from a smooth one, and it would be a new approach for surface roughness tactile discrimination as well. Furthermore, *S*(*FFT*), with an increasing normal load applied on the interface, presents six flat curves, as shown in Figure 4d, which shows unobvious changes in *S*(*FFT*) with an increasing normal load for all of the samples. As a result, the normal load will not affect the roughness tactile discrimination by changing the sliding velocity.

**Figure 5:**
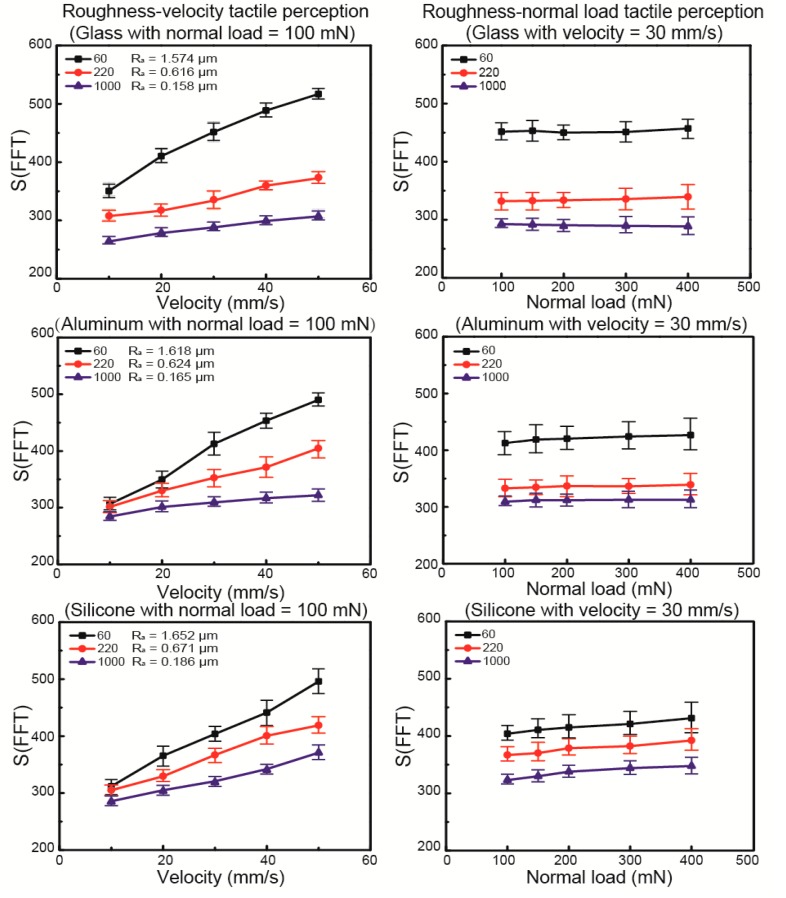
Glass, aluminum, and silicone roughness and hardness tactile perception as a function of velocity and normal load.

Glass, aluminum, and silicone samples with three different surface roughnesses were obtained by 60, 200, and 1000 mesh grinding to guarantee a group of samples with comparable surface roughness and various hardness. An *S*(*FFT*) analysis for each sample as a function of velocity and normal load was studied, as shown in Figure 5. It was found that, for the data of *S*(*FFT*), with an increasing normal load for each material, the slope of the curves, which was defined as *k_w_*, based on the least-square method, remained constant for the different surfaces’ roughness. However, it is also obvious that, for softer surfaces such as silicone, the *k_w_* is much larger, and for the data of *S*(*FFT*) with increasing velocity for each material, the slope of the curve *k_v_* increased for rougher surfaces. It can be inferred that *k_v_* and *k_w_* are independent from each other and related to the surface roughness and hardness, respectively.

### 3.3. Tactile Discrimination of PDMS with Different Hardness

**Figure 6:**
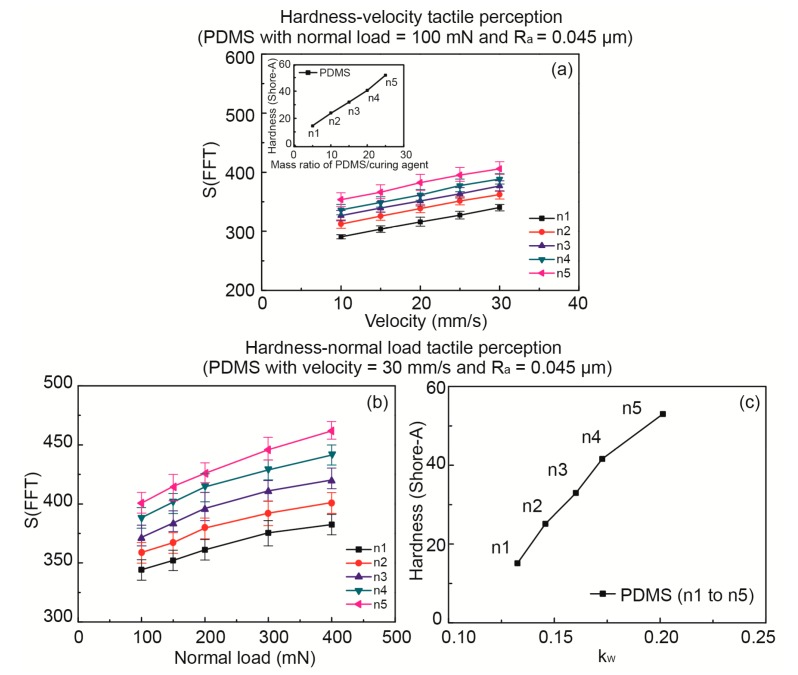
PDMS hardness tactile perception as a function of velocity and normal load. (**a**) Hardness-velocity tactile perception with the normal load equal to 100 mN; (**b**) Hardness-normal load tactile perception with velocity equal to 30 mm/s; (**c**) The correlation between PDMS hardness and *k_w_* values.

In the hardness tactile perception test, to further verify the *k_w_* value being able to distinguish material hardness, five PDMS samples were manufactured by different mass ratios of PDMS/curing agent (numbered from n1 to n5) to obtain five different hardnesses, and were tested under increasing velocity and normal load, respectively. All of the PDMS samples were identically patterned, and thus had the same surface roughness. The test was firstly taken with the scanning velocity varying from 10 to 30 mm/s and a fixed normal load of 100 mN. Then, it continued with the normal load varying from 100 to 400 mN and a fixed scanning velocity of 30 mm/s. The hardness of each sample was tested and is shown in the inset of Figure 6a, which shows an increasing hardness from n1 to n5. The *S*(*FFT*) with increasing velocity for the five samples is shown in Figure 6a, while the *S*(*FFT*) with an increasing normal load for the five samples is shown in Figure 6b. It is obvious that the slope of the curves in Figure 6a remains constant, which is related to the same roughness of the five samples while it increases. The relationship between the tested hardness and the obtained *k_w_* is shown in Figure 6c. It proves that *k_w_* could be a key parameter to distinguish material hardness, and the softer materials generated higher *k_w_* values.

### 3.4. Common Materials’ Roughness and Hardness Tactile Discrimination

**Figure 7:**
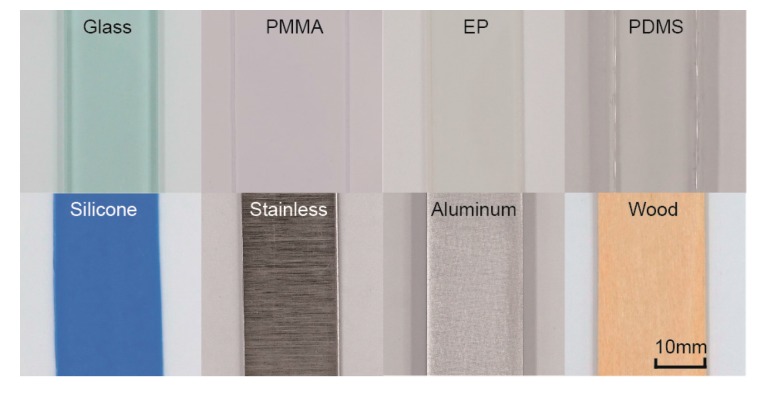
Schematic of eight common materials with various roughness and hardness.

**Figure 8:**
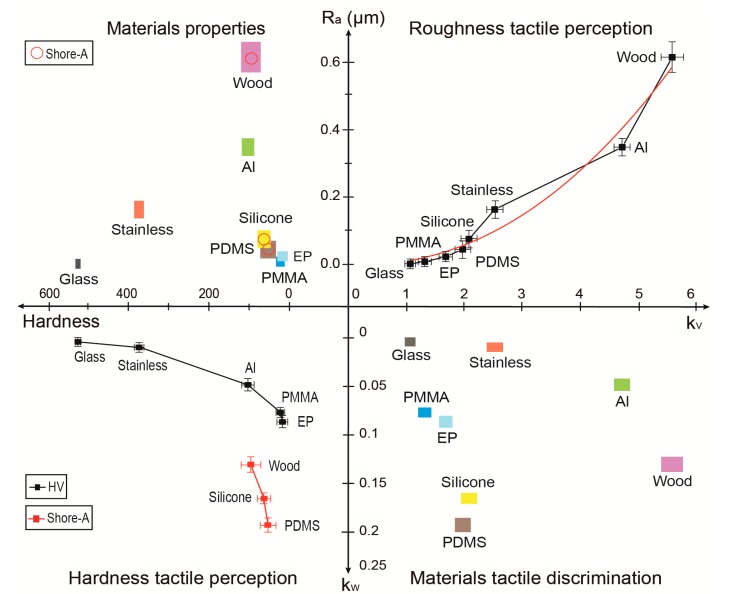
Cross-quadrant map of eight common materials’ tactile discrimination by traditional surface roughness, material hardness, and our new approach of *k_v_* and *k_w_* values.

In this section, eight common materials were tested and analyzed to carry out a new method of distinguishing materials by roughness and hardness tactile perception. The materials, such as common glass, PMMA, EP, PDMS, silicone, aluminum, and wood, as shown in Figure 7, were chosen as samples according to the variety of surface roughness and material hardness. The roughness and the hardness of all of the samples were first measured and then tested by an artificial fingertip with an increased scanning velocity and normal load. The measured roughness, hardness, *k_v_*, and *k_w_* of all of the samples are shown in Figure 8. The roughness and hardness parameters are listed in Table 2.

The first quadrant in Figure 8 shows the relationship between the measured R_a_ and the obtained *k_v_* for each sample. The sigmoidal-logistic fitting curve presents a non-linear relationship, which is counterevidence that tactile perception was not only influenced by surface roughness, but other factors, such as material hardness. The third quadrant shows the relationship between the measured hardness and the obtained *k_w_* for each sample: an increasing trend of *k_v_* can be obtained with increasing R_a_, while a decreasing trend of *k_w_* can be obtained with increasing hardness, which is in agreement with the previous tests.

The second quadrant in Figure 8 shows the measured roughness and hardness that can be used for distinguishing samples of different materials. The area covered by the error bars of the measured values of hardness and roughness means the points inside probably indicate a certain sample. However, it is obvious that some of these areas for each sample overlap with each other, such as silicone and PDMS, and PMMA and EP, which demonstrates the shortcoming of this material discrimination method based on roughness and hardness parameters: it cannot determine which sample it is if the data is in the superposed area. Furthermore, the hardness of soft materials, such as PDMS, silicone, and wood, were measured by a Shore-A hardness tester and marked with red rings, while all of the other materials’ hardnesses were obtained by Vickers hardness (HV). So, it is difficult to distinguish soft materials from hard ones according to a simple parameter. Thus, the traditional method of material discrimination has difficulty in distinguishing between two similar materials, which requires a great deal of effort and time as well.

In comparison, in the fourth quadrant of Figure 8, a new tactile discrimination method is performed by using the obtained values of *k_v_* and *k_w_*. It is obvious that, for these eight samples, all the areas covered by the error bars for each sample are separate from each other, which shows excellent performance for tactile discrimination. Furthermore, *k_w_* can be used to represent the hardness of all of these samples, including the hard ones and the soft ones, which is an obvious advantage as compared with the normal hardness measurements, which are different and incomparable for soft and hard materials.

## 4. Conclusions

In this paper, the tactile perception of various materials was investigated. Tactile discriminations of roughness and hardness are proposed with an artificial fingertip sliding on the surface of materials with various scanning velocities and normal loads. Friction-induced vibration signals were measured and the frequency spectral integral *S*(*FFT*) were calculated to determine *k_v_* and *k_w_* values as the changing rate of the *S*(*FFT*) with increasing sliding velocity and normal load, respectively. It was found that *k_v_* and *k_w_* are monotonically related to the roughness and hardness of the surface, respectively. Eight common materials of glass, stainless steel, aluminum, PMMA, EP, PDMS, silicone, and wood were investigated. The proposed method to determine the materials’ tactile discrimination based on the values of *k_v_* and *k_w_* is more accurate and efficient compared to the traditional material discrimination method based on the measurement of surface roughness and hardness.

This study provides an insight into material tactile discrimination by artificial fingertip sliding against a reciprocating surface at various sliding velocities and normal loads. Such studies should help in tactile sensor development and humanoid robot sensing technologies.

## Figures and Tables

**Table 1:** Properties of six sandpaper ground aluminum samples.

Mesh Number	60	120	220	400	800	1000
R_a_ (µm)	1.618	0.842	0.624	0.368	0.241	0.165
Hardness	103.6	103.2	102.6	102.1	102.1	102
*k_v_*	4.73	3.51	2.42	1.84	1.41	0.96
*k_w_*	0.061	0.041	0.053	0.062	0.048	0.063

**Table 2:** Properties of eight common flat material samples.

Name	Wood	Al	Stainless	Silicone	PDMS	EP	PMMA	Glass
R_a_ (µm)	0.615	0.368	0.163	0.075	0.045	0.023	0.008	0.002
Hardness	95.2	102.6	372.1	56.4	53.1	16.5	21.6	530.6
*k_v_*	5.57	4.71	2.52	2.08	1.97	1.68	1.32	1.07
*k_w_*	0.133	0.053	0.008	0.162	0.191	0.084	0.0751	0.0007

PDMS: polydimethylsiloxane; EP: epikote; PMMA: polymethyl methacrylate.
